# The relationships between glutathione, glutathione-S-transferase and cytotoxicity of platinum drugs and melphalan in eight human ovarian carcinoma cell lines.

**DOI:** 10.1038/bjc.1991.279

**Published:** 1991-08

**Authors:** P. Mistry, L. R. Kelland, G. Abel, S. Sidhar, K. R. Harrap

**Affiliations:** Drug Development Section, Institute of Cancer Research, Sutton, Surrey, UK.

## Abstract

The role of glutathione (GSH) and GSH-S-transferase (GST) activity in modulating the cytotoxicity of four platinum drugs and melphalan was evaluated in eight human ovarian carcinoma cell lines. The cell lines were established from solid and ascitic tumours from pretreated and untreated patients, and showed a wide spectrum of sensitivity to several platinum II and platinum IV drugs; cisplatin, carboplatin, CHIP and tetraplatin. Intracellular glutathione concentration measured in the cell lines showed a significant (P = 0.05) correlation with IC50 values for cisplatin (r = 0.91), carboplatin (r = 0.87) and CHIP (r = 0.88). The correlation between GSH levels and IC50 values for melphalan (r = 0.76) or tetraplatin (r = 0.60) was not as significant. GST activity showed no correlation with IC50 values, for the four platinum drugs. To determine the significance of the elevated GSH concentration in the refractory cell lines, the effect of D,L-buthionine-S, R-sulfoximine (BSO) mediated GSH depletion on platinum drug cytotoxicity was examined in one of the most sensitive (CH1) and two of the least sensitive (relatively resistant; SKOV-3, HX/62) cell lines. Comparison was made with the effect of GSH depletion on melphalan cytotoxicity in these three lines. These lines were differentially sensitive to BSO, with the two most platinum drug resistant lines being more tolerant to BSO than the sensitive CH1 line. Depletion of cellular GSH, ranging between 61 and 88%, had a differential effect on the sensitivity to PtII vs PtIV drugs in the three cell lines: cytotoxicity of the PtIV drugs, tetraplatin and CHIP, was substantially enhanced in both the resistant and sensitive cell lines; in contrast, the cytotoxicity of the PtII drugs, cisplatin and carboplatin, was only significantly increased in one of the two relatively resistant lines (SKOV-3) and in the sensitive (CH1) line after GSH depletion. Moreover the dose modification factor (DMF) for the PtII agents were lower than those for PtIV agents in the three cell lines. The dose modification factor for tetraplatin after BSO treatment was similar to that observed for melphalan in all three cell lines. In the SKOV-3 cell line extending the BSO pretreatment period to 48 h from 24 h marginally reduced the cytotoxicity of cisplatin, whereas the cytotoxicity of the other three drugs remained similar to that observed after 24 h BSO pretreatment. In contrast, extending the BSO treatment to 24 h after drug exposure potentiated the cytotoxicity of cisplatin, CHIP and tetraplatin.(ABSTRACT TRUNCATED AT 400 WORDS)


*Br. J. Cancer* (1991), **64**, 215–220 © Macmillan Press Ltd., 1991

### The relationships between glutathione, glutathione-S-transferase and cytotoxicity of platinum drugs and melphalan in eight human ovarian carcinoma cell lines

P. Mistry, L.R. Kelland, G. Abel, S. Sidhar & K.R. Harrap

*Drug Development Section, The Institute of Cancer Research, Belmont, Sutton, Surrey SM2 5NG, UK.*

**Summary** The role of glutathione (GSH) and GSH-S-transferase (GST) activity in modulating the cytotoxicity of four platinum drugs and melphalan was evaluated in eight human ovarian carcinoma cell lines. The cell lines were established from solid and ascitic tumours from pretreated and untreated patients, and showed a wide spectrum of sensitivity to several platinum II and platinum IV drugs; cisplatin, carboplatin, CHIP and tetraplatin. Intracellular glutathione concentration measured in the cell lines showed a significant ($P = < 0.05$) correlation with $IC_{50}$ values for cisplatin ($r = 0.91$), carboplatin ($r = 0.87$) and CHIP ($r = 0.88$). The correlation between GSH levels and $IC_{50}$ values for melphalan ($r = 0.76$) or tetraplatin ($r = 0.60$) was not as significant. GST activity showed no correlation with $IC_{50}$ values, for the four platinum drugs. To determine the significance of the elevated GSH concentration in the refractory cell lines, the effect of D,L-buthionine-S, R-sulfoximine (BSO) mediated GSH depletion on platinum drug cytotoxicity was examined in one of the most sensitive (CH1) and two of the least sensitive (relatively resistant; SKOV-3, HX/62) cell lines. Comparison was made with the effect of GSH depletion on melphalan cytotoxicity in these three lines. These lines were differentially sensitive to BSO, with the two most platinum drug resistant lines being more tolerant to BSO than the sensitive CH1 line. Depletion of cellular GSH, ranging between 61 and 88%, had a differential effect on the sensitivity to PtII *vs* PtIV drus in the three cell lines: cytotoxicity of the PtIV drugs, tetraplatin and CHIP, was substantially enhanced in both the resistant and sensitive cell lines; in contrast, the cytotoxicity of the PtII drugs, cisplatin and carboplatin, was only significantly increased in one of the two relatively resistant lines (SKOV-3) and in the sensitive (CH1) line after GSH depletion. Moreover the dose modification factor (DMF) for the PtII agents were lower than those for PtIV agents in the three cell lines. The dose modification factor for tetraplatin after BSO treatment was similar to that observed for melphalan in all three cell lines. In the SKOV-3 cell line extending the BSO pretreatment period to 48 h from 24 h marginally reduced the cytotoxicity of cisplatin, whereas the cytotoxicity of the other three drugs remained similar to that observed after 24 h BSO pretreatment. In contrast, extending the BSO treatment to 24 h after drug exposure potentiated the cytotoxicity of cisplatin, CHIP and tetraplatin. The significance of these results in relation to the role of GSH in the mechanism of action of PtII and PtIV drugs is discussed.

Cisplatin, and the markedly less nephrotoxic second generation analogue carboplatin, are exceptionally useful anticancer drugs particularly in the treatment of ovarian and testicular cancers (Calvert *et al.*, 1985; Peckham *et al.*, 1985; Wiltshaw & Carr, 1974). However, their efficacy is often compromised by the development of resistance after an initial response. Furthermore a number of tumours are unresponsive (intrinsically resistant) to these drugs. Clearly there is a need to develop a new generation of more clinically effective platinum-based drugs and/or to derive methods for modulating the sensitivity of tumours to the currently available drugs. To address these issues we have developed a range of human ovarian carcinoma cell lines and related xenografts, whose reponse to established platinum drugs reflects that seen clinically (Hills *et al.*, 1989; Harrap *et al.*, 1990). The biochemical mechanisms underlying platinum drug sensitivity/resistance in these models are being characterised. Several mechanisms have been postulated to be involved in platinum drug resistance in tumour cells, including decreased drug accumulation, increased inactivation through interaction with cellular thiols, reduced platinum-DNA adduct formation and enhanced repair of DNA lesions (for review see De Graeff *et al.*, 1988; Eastman & Richon, 1986; Andrews & Howell, 1990).

Glutathione (GSH), the major intracellular non-protein thiol, plays an important role in a number of cellular functions, including enzyme activity, membrane transport, DNA synthesis and inactivation of xenobiotics and reactive intermediates (Meister & Anderson, 1983). GSH and GSH-dependent enzymes are known to reduce the cytotoxic activity and hence cause resistance to alkylating agents and several other therapeutic agents (Arrick & Nathan, 1984; Hamilton *et al.*, 1989). Platinum is known to react avidly with sulphur ligands, hence elevated cellular GSH may reduce the cytotoxicities of platinum drugs. However, the evidence for the involvement of GSH and its dependent enzymes in platinum-drug resistance still remains equivocal. Both elevated and unaltered cellular GSH levels have been reported in murine and human cells, including ovarian carcinoma cells with acquired resistance to cisplatin (Andrews *et al.*, 1985; Hamilton *et al.*, 1985; Lewis *et al.*, 1988; Richon *et al.*, 1987; Teicher *et al.*, 1987). Moreover, reduction of cellular GSH by D,L-buthionine-S, R-sulfoximine (BSO) pretreatment has had variable effects on cisplatin sensitivity in resistant cells (Andrews *et al.*, 1985, 1988; Hamilton *et al.*, 1985). The role of GSH in modulating PtIV drug cytotoxicity has not been investigated extensively.

The present report describes our investigations into the role of GSH in modulating the cytotoxicity of platinum drugs in eight human ovarian carcinoma cell lines, established from solid and ascites tumours from pretreated and untreated patients. Levels of GSH and glutathione-S-transferase activity were determined in these cell lines and compared with the cytotoxic activity of several platinum II and IV chemotherapeutic agents: cisplatin [*cis*-diamminedichloroplatinum(II)], carboplatin [*cis*-diamminecyclobutane-1,1-dicarboxylatoplatinum(II)], CHIP (iproplatin) [*cis*-dichloro-*bis*-isopropylaminetranshydroxyplatinum(IV)] and tetraplatin [d,l-*trans*-tetrachloro-1,2-diaminocyclohexaneplatinum (IV) ]. We have also evaluated, in one sensitive and two relatively resistant cell lines, the effect of BSO-mediated depletion of cellular GSH on the cytotoxicity of these four platinum drugs and compared this with the effects on melphalan (L-phenylalanine mustard) cytotoxicity, since the latter has been reported to be markedly enhanced after GSH depletion (Hamilton *et al.*, 1989).

Correspondence: P. Mistry, Drug Development Section, The Institute of Cancer Research, Block E, 15 Cotswold Road, Belmont, Sutton, Surrey SM2 5NG, UK.
Received 3 September 1990; and in revised form 19 March 1991.

### Materials and methods

*Chemicals*

Glutathione reductase (type IV bakers' yeast), GSH, 5,5'-dithiobis-2- (nitrobenzoic acid) (DTNB), D,L-buthionine-S, R-sulfoximine and 5-sulfosalicylic acid were purchased from Sigma Chemicals UK Ltd. 1-Chloro-2,4-dinitrobenzene (CDNB) was obtained from Aldrich Chemical Co Ltd, Dorset. Cisplatin, carboplatin and CHIP were obtained from the Johnson Matthey Technology Centre. Tetraplatin was a gift from Dr M. Wolpert-Defilippes (NCI, Bethesda, MD, USA).

*Cell lines*

Eight human ovarian carcinoma cell lines were used in this study. Six (SKOV-3, HX/62, PXN/94, OVCAR-3, CH1 and 41M) have been described in detail previously (Hills *et al.*, 1989). The new lines, LK1 and LK2, were established from ascitic fluid using methods as described previously (Hills *et al.*, 1989). The patient's pre-biopsy treatment and response were as follows: LK1, carboplatin with partial response and trimelamol with stable disease; LK2, carboplatin with no response and cisplatin with no response.

*Drug exposure and cytotoxicity assay*

Cells were grown in Dulbecco's Modified Eagles Medium (DMEM) plus 10% foetal calf serum, 50 $\mu g\ ml^{-1}$ gentamicin, 2.5 $\mu g\ ml^{-1}$ amphotericin B, 2 mM glutamine, 10 $\mu g\ ml^{-1}$ insulin and 0.5 $\mu g\ ml^{-1}$ hydrocortisone. Cells were periodically checked and found to be free of mycoplasma and used in these studies from passage 25 to 50. The sensitivity of each cell line to the four platinum agents (cisplatin, carboplatin, CHIP and tetraplatin) was examined as described previously (Hills *et al.*, 1989) with the following modifications: single cells harvested by trypsinisation (0.02% EDTA/0.05% trypsin) were plated at between $5 \times 10^3$ and $1 \times 10^4$ in 96-well microtitre plates. After allowing attachment overnight, cells were exposed to agents in quadruplicate wells for a total of either 2 or 96 h. At the end of the 2 h period fresh medium was applied and the cells were grown for a further 96 h. Cytotoxicity was then assessed by staining basic amino acids with sulforhodamine B (SRB), as modified from Skehan *et al.* (1989). Briefly, the medium was aspirated and the cells exposed to ice-cold 10% w/v trichloroacetic acid (TCA) for 30 min. For cell lines prone to detachment (CH1, LK2) from the wells, a 5 min bath in ice-cold methanol was then included. Cells were then washed five times with tap water, held in 100 $\mu l$ 0.4% SRB (Sigma Chemicals) in 1% acetic acid for 10 to 15 min and washed five times with 1% acetic acid. After air-drying overnight, the protein bound SRB was solubilised with 100 $\mu l$ of 10 mM Tris base and the plates read at 540 nm using a plate reader (Titertek Multiscan MCC/340, Flow Laboratories). By comparing treated with untreated control wells, $IC_{50}$ values were then determined using a computer software package (Titersoft II, Flow Laboratories).

*GSH assay*

Cellular GSH content was determined in cells grown under conditions identical to those used for cytotoxicity assay. For adequate cell numbers, however, $0.5-1.0 \times 10^6$ cells were plated as monolayers in T25 flasks (Flow Laboratories). After a 24 h attachment period the medium was aspirated and the cells were washed twice in 10 ml of cold phosphate buffered saline (PBS), pH 7.4. Cellular GSH was then extracted according to Russo *et al.* (1986), by addition of 2.0 ml of cold 0.6% sulphosalicylic acid followed by 10 min incubation at 4°C with occasional shaking. Total GSH in the extract was assayed by the method of Griffiths (1980). The protein content of the extracted cells was analysed according to Lowry *et al.* (1951) after solubilisation in 2.0 ml of 1.0 N sodium hydroxide. The GSH content was expressed as nmol per $10^6$ cells or per mg protein.

*Effect of BSO exposure on intracellular GSH concentration*

Preliminary studies were performed to establish the effect of BSO treatment on intracellular GSH content in one sensitive (CH1) and two resistant (SKOV-3 and HX/62) cell lines. Single cells ($5 \times 10^5$) were plated in T25 flasks and divided into four groups of three flasks. After a 24 h attachment period, the medium was aspirated and replaced with medium containing BSO (2 groups) or vehicle (2 control groups). The BSO concentration ranged from 12.5 $\mu M$ to 50 $\mu M$, depending on the cell line investigated. One control and one BSO treated group were incubated for 24 h and the remainder were incubated for 48 h. At the end of these periods one flask from each group was used for cell count and the other two were used for GSH determination. Cellular GSH was extracted and analysed as described above. However, GSH was extracted using 1.0 ml of 0.6% sulphosalicylic acid in order to concentrate the extracts. Preliminary experiments had shown that maximum extraction was achieved with this volume. Cell growth and viability 96 h after exposure to BSO at each concentration and time period was also examined in a separate experiment. The BSO concentration and time of exposure which did not alter cell growth and viability were subsequently used to examine the effect of cellular GSH depletion on platinum-drug sensitivity. These values were as follows: 50 $\mu M$ BSO, 24 h exposure for the two resistant lines, SKOV-3 and HX/62 and 12.5 $\mu M$ BSO, 24 h exposure for the sensitive line, CH1.

*Effect of cellular GSH depletion on the cytotoxicity of platinum drugs*

The effect of glutathione depletion on cellular response to platinum drugs was assessed in three cell lines, SKOV-3, HX/62 and CH1 as described above with the following modification. Cells seeded in 96-well microtitre plates were allowed to attach overnight and then incubated in a medium containing an appropriate concentration of BSO or vehicle for a further 24 h. This was followed by a 2 h incubation in medium containing appropriate concentrations of platinum drug and BSO or vehicle. At the end of this period fresh medium was applied and cell survival assessed 96 h later as described above.

*Glutathione-S-transferase (GST) assay*

Following a 24 h attachment period in T25 flasks, cells ($3-5 \times 10^6$) in log phase growth were washed twice with 20 ml of cold PBS, scraped and harvested using 3.0 ml of PBS. The cell suspension was sonicated (Polytron sonicator, MSE, Fisons Ltd) using $3 \times 5$ s pulses at 0.75 max power with 20 s cooling period at 4°C between each pulse. The cell sonicate was centrifuged at 11,000 *g* for 25 min at 4°C and the supernatant analysed for GST activity using 1 mM CDNB as the substrate (Habig *et al.*, 1974).

### Results

*Cytotoxicity of platinum drugs*

Cytotoxicity of cisplatin, carboplatin, CHIP and tetraplatin in the eight human ovarian carcinoma cell lines is shown in Figure 1. The cell lines exhibited a wide range of sensitivity to the four platinum compounds. The HX/62 and SKOV-3 cells lines were generally the most resistant, while the PXN/94 cell line showed differential sensitivity to the four platinum agents, being very sensitive to tetraplatin relative to the other three drugs.

**Figure 1** Sensitivity of the eight human ovarian carcinoma cell lines to the four platinum drugs. $IC_{50}$ values (mean ± s.d., $n = 4-7$) were assessed by sulforhodamine B assay using a 96 h continuous drug exposure as outlined in Materials and methods.

*Relationship between GSH concentration and $IC_{50}$ values of platinum drugs and melphalan*

The concentration of GSH and GST activity in the eight ovarian carcinoma cell lines is shown in Table I. A significant correlation was observed between intracellular GSH concentration (expressed as either nmol per $10^6$ cells or per mg protein) and $IC_{50}$ values obtained after 2 or 96 h continuous exposure to cisplatin ($r = 0.91$), carboplatin ($r = 0.87$) and CHIP ($r = 0.88$) in these cell lines (Table II and Figure 2a–c). The GSH content did not correlate with sensitivity to tetraplatin when all eight cell lines were considered ($r = 0.13$). However, without the inclusion of the PXN/94 cell line, which is differentially sensitivity to tetraplatin, and which also contains high GSH concentration the correlation ($r = 0.60$) was improved (Table II and Figure 2d). The mechanisms involved in the exquisite sensitivity of the PXN/94 cell line to tetraplatin is currently being investigated.

The $IC_{50}$ values for melphalan also showed a positive correlation ($r = 0.77$) with cellular GSH content in six of the cell lines. However, statistical significance was not achieved (Table II).

*The effect of BSO on cellular GSH concentration*

Treatment of SKOV-3 cells with 25 μM BSO reduced the GSH levels by 70% and 83% at 24 h and 48 h, respectively. Exposure to 50 μM BSO reduced the GSH concentration by 88% and 97% respectively, at the two time points (Figure 3a). Furthermore no growth delay or loss of viabilty were observed 96 h after exposure to 50 μM BSO up to 48 h. In another resistant cell line, HX/62, treatment with 50 μM BSO for 24 h, which reduced cellular GSH content by 61% also had no effect on cell growth and viability (Figure 3b). However, exposure to 50 μM BSO for 48 h did partially reduce the growth rate of these cells. In contrast, in the CH1 cell line, which is one of the most sensitive to platinum drugs, exposure to BSO was less well tolerated than in the resistant cell lines. Exposure of CH1 cells to 25 μM BSO for 24 h had an adverse effect on cell viability, although exposure to 12.5 μM BSO for 24 h, which reduced cellular GSH by 81%, was well tolerated (Figure 3c).

*Effect of BSO pretreatment on cytotoxicity of platinum-drugs and melphalan*

Reduction of cellular GSH by 88% in the SKOV-3 cells significantly ($P < 0.01$) potentiated the cytotoxicity of all four Pt drugs. However, the dose modification factor (DMF) for the PtIV drugs, CHIP and tetraplatin was greater than for the PtII drugs, cisplatin and carboplatin (Table III). In the HX/62 cell line a 61% reduction in its GSH content did not potentiate the cytotoxicity of PtII drugs, whereas the sensitivity to PtIV drugs was significantly enhanced (Table III). In both SKOV-3 and HX/62 cell lines the DMF for tetra-

**Table I** GSH levels and GST-activity in the eight human ovarian carcinoma cell lines

| Cell line | *GSH concentration*[a] *nmol $10^{-6}$ cells* | *nmol $mg^{-1}$ protein* | *n*[b] | *GST specific activity*[c] *nmol product $min^{-1}$ $mg^{-1}$ protein* |
|---|---|---|---|---|
| HX/62 | 59.8±8.7 | 47.0±7.8 | 8 | 109±21 |
| SKOV-3 | 50.2±6.0 | 72.3±16.0 | 8 | 150±14 |
| PXN/94 | 60.2±18.1 | 59.3±18.8 | 11 | 145±22 |
| OVCAR-3 | 20.4±7.6 | 26.4±3.4 | 9 | 199±25 |
| 41M | 8.8±0.6 | 18.2±2.5 | 10 | 135±18 |
| LK1 | 13.3±0.4 | 31.8±1.2 | 3 | 115 |
| LK2 | 19.0±0.3 | 31.1±0.3 | 3 | 155 |
| CH1 | 19.4±3.0 | 24.2±3.6 | 10 | 122±6 |

[a]Values are mean ± s.d. [b]Number of determinations on at least four separate cultures except for LK1 and LK2 cell lines, where all the samples were from one culture. [c]GST activity mean ± s.d. ($n$ = four separate cultures, except for LK1 and LK2 cell lines where $n = 2$).

**Table II** Correlation coefficients ($r$) between intracellular GSH content and $IC_{50}$ values for the four platinum drugs and melphalan in eight human ovarian carcinoma cell lines

| | *Correlation coefficient (r)*[a] | | | |
|---|---|---|---|---|
| *Drug* | *GSH nmol $10^{-6}$ cells with 2 h $IC_{50}$* | *96 h $IC_{50}$* | *GSH nmol $mg^{-1}$ protein with 2 h $Ic_{50}$* | *96 h $IC_{50}$* |
| Cisplatin | 0.83 | 0.91 | 0.69 | 0.78 |
| *P* | <0.03 | <0.005 | NS | <0.03 |
| Carboplatin | 0.85 | 0.87 | 0.69 | 0.75 |
| *P* | <0.01 | <0.05 | NS | <0.05 |
| CHIP | 0.87 | 0.88 | 0.65 | 0.73 |
| *P* | <0.01 | <0.005 | NS | <0.05 |
| Tetraplatin | 0.18 (0.69)[b] | 0.13 (0.60) | 0.39 (0.85) | 0.34 (0.77) |
| *P* | NS (NS) | NS (NS) | NS (<0.03) | NS (<0.05) |
| Melphalan[c] | – | 0.76 | – | 0.71 |
| *P* | | NS | | (NS) |

[a]Correlation coefficients were determined from linear regression analysis. [b]Figure in parentheses represent value calculated without the inclusion of the PXN/94 cell line. [c]Correlation coefficient determined using 96 h $IC_{50}$ values from six cell lines; cytotoxicities of melphalan in the LK1 and LK2 cell lines were not determined.

**Figure 2** Correlation between intracellular glutathione content and sensitivity of the ovarian cell lines to four platinum drugs. $IC_{50}$ values were determined using a 96 h continuous drug exposure assay as outlined in Materials and methods. In panels **a** and **b** points for two lines, CH1 and LK2, overlap. In panel **d**, dotted line excludes PXN/94 cell line data. s.d. (bars) was smaller than the symbol size where not indicated.

**Figure 3** Effect of BSO treatment time on intracellular GSH concentration in **a**, SKOV-3, **b**, HX/62 and **c** CH1 cell lines. GSH levels were determined as stated in Materials and methods. Open bars represent control cells and dashed bars represent BSO-treated cells. The CH1 cells were treated with 12.5 μM BSO and the SKOV-3 and HX/62 cells were treated with 50 μM BSO. Values are mean ± s.d. where $n = 4$ except for HX/62 48 h time point where $n = 2$.

platin was similar to that observed for melphalan. An 81% depletion of intracellular GSH in the sensitive CH1 cells caused a significant potentiation of PtII drug cytotoxicity. Moreover, the DMFs for these drugs were greater than those observed in the resistant lines (Table III). Tetraplatin and melphalan cytotoxicity in the CH1 cells was enhanced to a similar extent as that seen in the resistant cells after GSH depletion (Table III). In preliminary experiments with SKOV-3 cells we observed that extending the BSO pretreatment period from 24 h to 48 h before exposure to the drugs marginally reduced the DMF for cisplatin from 1.25–1.33 (range, $n = 2$) to 0.72–0.92 (range, $n = 2$), whereas the DMF for carboplatin, CHIP and tetraplatin remained similar in the two groups. In addition, exposure of the SKOV-3 cells to 50 μM BSO for 24 h prior to and following drug treatment increased the DMF for cisplatin, CHIP and tetraplatin relative to that observed after 24 h BSO pretreatment only (Table IV). In contrast, the DMF for carboplatin in these two treatment groups was similar.

*Intracellular GST concentrations*

The GST activity in the eight cell lines, measured using 1-chloro-2, 4-dinitrobenzene as the substrate, are shown in Table I. No correlation was observed between GST activity and $IC_{50}$ values for any of the four platinum drugs examined.

### Discussion

In this study we have measured GSH levels in eight human ovarian carcinoma cell lines established from patients who were either untreated or pretreated with platinum and non-platinum-containing regimens. This panel of lines had a range of sensitivity to platinum drugs similar to that observed in the clinic (Hills *et al.*, 1989); with the SKOV-3 and HX/62 cells lines being the most refractory (relatively resistant), and the CH1 line being one of the most sensitive. Cellular GSH was determined in cells grown under conditions reflecting those used for drug cytotoxicity assay, because GSH and GSH-dependent enzyme levels vary with time after plating in both murine and human cells (Batist *et al.*, 1986; Post *et al.*, 1983). Indeed, in agreement with these reports, our own data showed that GSH levels per $10^6$ cells were higher at 24 h than at 48 h after plating. The GSH concentrations in the eight ovarian cell lines showed a strong inverse correlation with sensitivity to both PtII and PtIV group drugs. As far as we are aware this is the first time such a correlation has been observed in a group of human ovarian carcinoma cells with such a wide (approximately 100-fold) range of sensitivity to platinum drugs (Hills *et al.*, 1989). Recently Hosking *et al.* (1990) have reported a correlation between cisplatin sensitivity in various cell types and their GSH content. Elevated GSH levels in human cells with acquired resistance to cisplatin have also been reported (Hamilton *et al.*, 1985; Lewis *et al.*, 1988; Teicher *et al.*, 1987). In one study, Lewis *et al.* (1988) found that, relative to a sensitive ovarian cell line (PE01), GSH and GSH-dependent enzymes were higher in a resistant (PE04) line established from the same patient after relapse on cisplatin-containing therapy.

To establish whether the elevated GSH levels played a role in the mechanism of resistance to platinum drugs we examined the effect of BSO- mediated GSH depletion on the cytotoxicity of these drugs in one sensitive (CH1) and two relatively resistant (SKOV-3, HX/62) cell lines. The cell lines were found to be differentially sensitive to BSO exposure, with the most sensitive being the CH1 cell line, followed by HX/62 and then SKOV-3; hence the platinum resistant cells were more tolerant to BSO than the platinum sensitive cells. Depletion of cellular GSH had a differential effect on the cytotoxicity of PtII *vs* PtIV complexes in these three cell lines. The cytotoxicity of PtIV drugs was enhanced significantly ($P < 0.05$) in all three cell lines whereas the cytotoxicity of the PtII drugs was only significantly enhanced in one

**Table III** Dose modification factors for platinum drugs and melphalan in human ovarian carcinoma cell lines following BSO-mediated GSH reduction

| *Cell line* | *Cisplatin* | *Carboplatin* | *DMF* CHIP* | *Tetraplatin* | *Melphalan* |
|---|---|---|---|---|---|
| SKOV-3 | 1.34±0.26 | 1.16±0.11 | 1.61±0.18 | 2.31±0.91 | 2.74±0.21 |
| *n* | 11 | 10 | 10 | 10 | 3 |
| *P*† | <0.01 | <0.01 | <0.05 | <0.01 | <0.01 |
| HX/62 | 1.26±0.29 | 0.91±0.15 | 1.67±0.04 | 2.89±1.35 | 3.04±1.05 |
| *n* | 4 | 4 | 4 | 4 | 4 |
| *P* | NS | NS | <0.01 | <0.05 | <0.05 |
| CH1 | 1.49±0.50 | 1.52±0.38 | 1.52±0.39 | 2.55±2.09 | 2.75±1.43 |
| *n* | 7 | 6 | 5 | 6 | 5 |
| *P* | <0.05 | <0.05 | <0.05 | <0.05 | <0.01 |

*DMF = ($IC_{50}$ in the absence of BSO)/($IC_{50}$ for drug after BSO pretreatment), values are mean ± s.d. Cellular GSH in the SKOV-3, HX/62 and CH1 cell lines was depleted by 88, 61 and 81%, respectively. †Statisticaly significance was tested by paired *t*-test.

**Table IV** Effect of BSO treatment period on dose modification factors for platinum drugs in the SKOV-3 cell line

| *BSO treatment[b] period* | *Cisplatin* | *Carboplatin* | *DMF[a] CHIP* | *Tetraplatin* |
|---|---|---|---|---|
| 24 h pre-drug drug exposure | 1.55 (1.32–1.74) | 1.28 (1.25–1.30) | 1.41 (1.28–1.60) | 1.75 (1.63–1.85) |
| 24 h per- and 24 h post-drug exposure | 1.98 (1.53–2.24) | 1.32 (1.21–1.38) | 2.62 (2.33–2.95) | 2.10 (1.63–2.40) |

[a]DMF = ($IC_{50}$ in the absence of BSO)/($IC_{50}$ for drug after BSO treatment). Values reported are mean (range in parentheses) of three experiments. [b]Time of exposure to 50 μM BSO in relation to 2 h drug exposure; BSO was also present during exposure to the drugs.

of the resistant (SKOV-3; $P<0.01$) and in the sensitive (CH1; $P<0.05$) cell line. Moreover, of the four platinum drugs examined the highest DMF was observed for tetraplatin followed by CHIP in the three cell lines. Hence BSO mediated GSH depletion acts as a sensitiser of PtIV drug action in the sensitive as well as in the relatively resistant cells. A substantial increase in cytotoxicity of CHIP but not that of cisplatin or carboplatin in two murine cell lines after GSH depletion have also been reported (Brock & Smith, 1988). However, Andrews *et al.* (1985) showed no alteration in sensitivity to CHIP or PtII drugs after GSH depletion in human ovarian cell lines with acquired resistance to cisplatin. These authors subsequently reported (Andrews *et al.*, 1988) that cisplatin resistance in these cells could be partially reversed if GSH depletion was maintained after drug treatment by prolonging the exposure to BSO. Maintenance of BSO exposure during cisplatin treatment also increased drug cytotoxicity in both sensitive and resistant A2780 ovarian carcinoma cell lines (Hamilton *et al.*, 1985). Our results also showed that, in the relatively resistant SKOV-3 cell line, maintenance of GSH depletion 24 h after drug treatment increased the DMF for cisplatin, CHIP and tetraplatin relative to that observed when cells were pretreated with BSO for 24 h only. This increase may be due to inhibition of DNA repair as well as reduced inactivation of active species as a result of GSH depletion (Lai *et al.*, 1989).

The fact that the DMF for PtIV drugs were greater than those for PtII drugs in the relatively resistant cells suggests that GSH may play a more significant role in the mechanism of resistance to PtIV than PtII drugs. However, it is possible that BSO may have interfered with PtII drug action, particularly that of cisplatin, in the two resistant cell lines either directly or indirectly through a mechanism which counteracts the beneficial effects of GSH depletion. Evidence to support this comes from the fact that the DMFs for cisplatin after 24 h BSO exposure in the resistant cells were marginally higher than those observed after 48 h BSO treatment. In addition, cisplatin induced nephrotoxicity has been shown to be augmented by concomitant administration of BSO (Mayer *et al.*, 1987). The differential sensitisation of the resistant ovarian cells to PtII and PtIV drugs after BSO pretreatment may be due to the differences in the rate at which GST catalyses the reaction of these complexes with GSH. The relationship between GST activity and platinum drug action is unclear and both increased and unaltered activity have been reported in cells with acquired resistance to cisplatin (Hamilton *et al.*, 1985; Lewis *et al.*, 1988; Wang *et al.*, 1989). Our own data showed no correlation between total GST activity and sensitivity to the four platinum drugs. However, it is possible that a specific isoenzyme may mediate a differential response, and GST isoenzyme profiles are currently being investigated in these cell lines. The fact that the DMFs for tetraplatin and the PtII drugs are different after GSH depletion is intriguing because the complex entering the ovarian cells after exposure to tetraplatin is probably 1,2-diaminocyclohexanedichloroplatinum (II), since tetraplatin is rapidly reduced to this complex ($t_{\frac{1}{2}}$, 5–15 min) in RPMI tissue culture medium and plasma (Gibbons *et al.*, 1989). Our own data (unpublished) have also shown a very rapid reduction of tetraplatin by the ovarian cell culture medium. Moreover, the enhancement of PtIV drug cytotoxicity after GSH depletion is interesting since it is widely believed that these drugs are activated by reduction to PtII complexes and that this process is partially mediated by intracellular GSH (Cleare, 1977; Eastman, 1987). On this basis GSH depletion should have antagonised PtIV drug cytotoxicity.

Our data confirm that melphalan cytotoxicity is greatly enhanced by GSH depletion in both sensitive and resistant ovarian carcinoma cell lines (Andrews *et al.*, 1985; Green *et al.*, 1984; Hamilton *et al.*, 1985). Moreover the DMF values observed for tetraplatin were similar to those observed for melphalan in the three cell lines examined. If the enhanced cytotoxicity of tetraplatin and CHIP after GSH depeletion represents a typical response of PtIV complexes then this could be of significance in future platinum drug development.

In summary, intracellular GSH but not GST levels showed a strong inverse correlation with sensitivity to PtII and PtIV drugs in eight human ovarian carcinoma cell lines. However, modulation of platinum drug cytotoxicity in response to BSO pretreatment suggests that GSH may play a more significant role in the mechanism of resistance to PtIV than PtII drugs. The mechanisms involved in this differential response are unknown and need to be investigated further along with the intracellular metabolism of these complexes in order to evaluate the exact role of GSH and GSH-dependent enzymes in modulating platinum drug action.

This work was supported by grants to The Institute of Cancer Research: Royal Cancer Hospital from the Cancer Research Campaign and the Medical Research Council, The Johnson Matthey Technology Centre and Bristol-Myers Squibb. The authors would like to thank Mrs A. Ford and Miss A. Pritchard for the efficient typing of this manuscript.

### References

ANDREWS, P.A., MURPHY, M.P. & HOWELL, S.B. (1985). Differential potentiation of alkylating and platinating agent cytotoxicity in human ovarian carcinoma cells by glutathione depletion. *Cancer Res.*, **45**, 6250.

ANDREWS, P.A., SCHIEFER, M.A., MURPHY, M.P. & HOWELL, S.B. (1988). Enhanced potentiation of cisplatin cytotoxicity in human ovarian carcinoma cells by prolonged glutathione depletion. *Chem.-Biol. Interact.*, **65**, 51.

ANDREWS, P.A. & HOWELL, S.B. (1990). Cellular pharmacology of cisplatin: perspectives on mechanisms of acquired resistance. *Cancer Cells*, **2**, 35.

ARRICK, B.A. & NATHAN, C.F. (1984). Glutathione as a determinant of therapeutic efficacy: a review. *Cancer Res.*, **44**, 4224.

BATIST, G., BEHRENS, B.C., MAKUCH, R. & 5 others (1986). Serial determination of glutathione levels and glutathione-related enzyme activies in human tumour cells *in vitro*. *Biochem. Pharmacol.*, **35**, 2257.

CALVERT, A.H., HARLAND, S.J., NEWELL, D.R., SIDDIK, Z.H. & HARRAP, K.R. (1985). Phase I studies with carboplatin at the Royal Marsden Hospital. *Cancer Treat. Rev.*, **12** (Suppl. A), 51.

CLEARE, M. (1977). Some aspects of platinum complex chemistry and their relation to antitumour activity. *J. Clin. Hematol. Oncol.*, **7**, 1.

DE GRAEFF, A., SLEBOS, R.J. & RODENHUIS, S. (1988). Resistance to cisplatin and analogues: mechanisms and potential clinical implications. *Cancer Chemother. Pharmacol.*, **22**, 325.

EASTMAN, A. (1987). Glutathione-mediated activation of anticancer platinum (IV) complexes. *Biochem. Pharmacol.*, **36**, 4177.

EASTMAN, A. & RICHON, V.M. (1986). In *Biochemical Mechanisms of Platinum Antitumour Drugs*, McBrien, D.C.H. & Slater, T.F. (eds). p. 91. IRL Press Ltd: Oxford.

GIBBONS, G.P., WYRICK, S. & CHANEY, S.G. (1989). Rapid reduction of tetrachloro (D,L-trans) 1,2-diaminocyclohexane platinum IV (tetraplatin) in RPMI 1640 tissue culture medium. *Cancer Res.*, **49**, 1402.

GREEN, J.A., VISTICA, R.C., YOUNG, R.C., HAMILTON, T.C., ROGAN, A.M. & OZOLS, R.F. (1984). Potentiation of melphalan cytotoxicity in human ovarian cell lines by glutathione depletion. *Cancer Res.*, **44**, 5427.

GRIFFITHS, O.W. (1980). Determination of glutathione and glutathione disulfide using glutathione reductase and 2-vinyl pryidine. *Analytical Biochem.*, **106**, 207.

HABIG, W.H., PABST, M.J. & JAKOBY, W.B. (1974). Glutathione S-transferases – the first enzymatic step in mercapturic acid formation. *J. Biol. Chem.*, **249**, 7130.

HAMILTON, T.C., WINKER, M.A., LOUIE, K.G. & 7 others (1985). Augmentation of adriamycin, melphalan and cisplatin cytotoxicity in drug-resistant and -sensitive human ovarian carcinoma cell lines by buthionine sulfoximine mediated glutathione depletion. *Biochem. Pharamcol.*, **34**, 2583.

HAMILTON, T.C., LAI, G.-M., ROTHENBERG, M.L., FOJO, A.T., YOUNG, R.L. & OZOLS, R.F. (1989). In *Drug Resistance in Cancer Therapy*, Ozols, R.F. (ed.) p. 151. Kluwer Academic Publishers: Boston.

HARRAP, K.R., JONES, M., SIRACKY, J., POLLARD, L.A. & KELLAND, L.R. (1990). The establishment, characterization and calibration of human ovarian carcinoma xenografts for the evaluation of novel platinum anticancer drugs. *Annals Oncol.*, **1**, 65.

HILLS, C.A., KELLAND, L.R., ABEL, G., SIRACKY, J., WILSON, A.P. & HARRAP, K.R. (1989). Biological properties of ten human ovarian carcinoma cell lines: calibration *in vitro* against four platinum complexes. *Br. J. Cancer*, **59**, 527.

HOSKING, L.K., WHELAN, R.D., SHELLARD, S.A., BEDFORD, P. & HILL, B.T. (1990). An evaluation of the role of glutathione and its associated enzymes in the expression of differential sensitivities to antitumour agent shown by a range of human tumour cell lines. *Biochem. Pharmacol.*, **40**, 1833.

LAI, G-M., OZOLS, R.F., YOUNG, R.C. & HAMILTON, T.C. (1989). Effect of Glutathione on DNA repair in cisplatin-resistant human ovarian cancer cell lines. *J. Natl Cancer Inst.*, **81**, 535.

LEWIS, A.D., HAYES, J.D. & WOLF, C.R. (1988). Glutathione and glutathione-dependent enzymes in ovarian adenocarcinoma cell lines derived from a patient before and after the onset of drug resistance: intrinsic differences and cell cycle effects. *Carcinogenesis*, **9**, 1283.

LOWRY, O.H., ROSEBROUGH, M.T., FARR, A.L. & RANDALL, R.J. (1951). Protein measurements with the folin phenol reagent. *J Biol. Chem.*, **193**, 265.

MAYER, R.D., LEE, K. & COCKETT, T.K. (1987). Inhibition of cisplatin-induced nephrotoxicity in rats by buthionine sulfoximine, a glutathione synthesis inhibitor. *Cancer Chemother. Pharmacol.*, **20**, 207.

MEISTER, A. & ANDERSON, M.E. (1983). Glutathione. *Ann. Rev. Biochem.*, **52**, 711.

PECKHAM, M.J., HORWICH, A., BRADA, M., DRURY, A. & HENDRY, W.F. (1985). Cis-diammine-1,1-cyclobutane dicarboxylate platinum II (carboplatin) in the treatment of testicular germ cell tumours: a preliminary report. *Cancer Treat. Rev.*, **12** (Suppl. A), 101.

POST, G.B., KELLER, D.A., CONNOR, K.A. & MENZIEL, D.B. (1983). Effects of culture conditions on glutathione content in A549 cells. *Biochem. Biophys. Res. Commun.*, **114**, 737.

RICHON, V.M., SCHULTE, N. & EASTMAN, A. (1987). Multiple mechanisms of resistance to cis-diammine dichloro platinum (II) in murine leukaemia L1210 cells. *Cancer Res.*, **47**, 2056.

RUSSO, A., DE GRAFF, W., FRIEDMAN, N. & MITCHELL, J.B. (1986). Selective modulation of glutathione levels in human normal versus tumour cells and subsequent differential response to chemotherapy drugs. *Cancer Res.*, **46**, 2845.

SKEHAN, P., STORENG, R., SCUDEIRO, N. & 7 others (1989). Evaluation of colorimetric protein and biomass stains for assaying *in vitro* drugs effects upon human tumour cell lines. *Proc. Am. Assoc. Cancer Res.*, **30**, 612.

SMITH, E. & BROCK, A.P. (1988). An *in vitro* study comparing the cytotoxicity of three platinum complexes with regard to the effect of thiol depletion. *Br. J. Cancer*, **57**, 548.

TEICHER, B.A., HOLDEN, S.A., KELLEY, M.J. & 5 others (1987). Characterisation of a human squamous carcinoma cell line resistant to cis-diammine dichloro platinum (II). *Cancer Res.*, **47**, 338.

WANG, Y., TEICHER, B.A., SHEA, T.C. & 4 others (1989). Cross-resistance and glutathione-S-transferase – levels among four human melanoma cell lines selected for alkylating agent resistance. *Cancer Res.*, **49**, 6185.

WILTSHAW, E. & CARR, B. (1974). In *Recent Results in Cancer Research*, Connors, T.A. & Roberts, J.J. (eds), p. 178. Springer Verlag: Berlin.


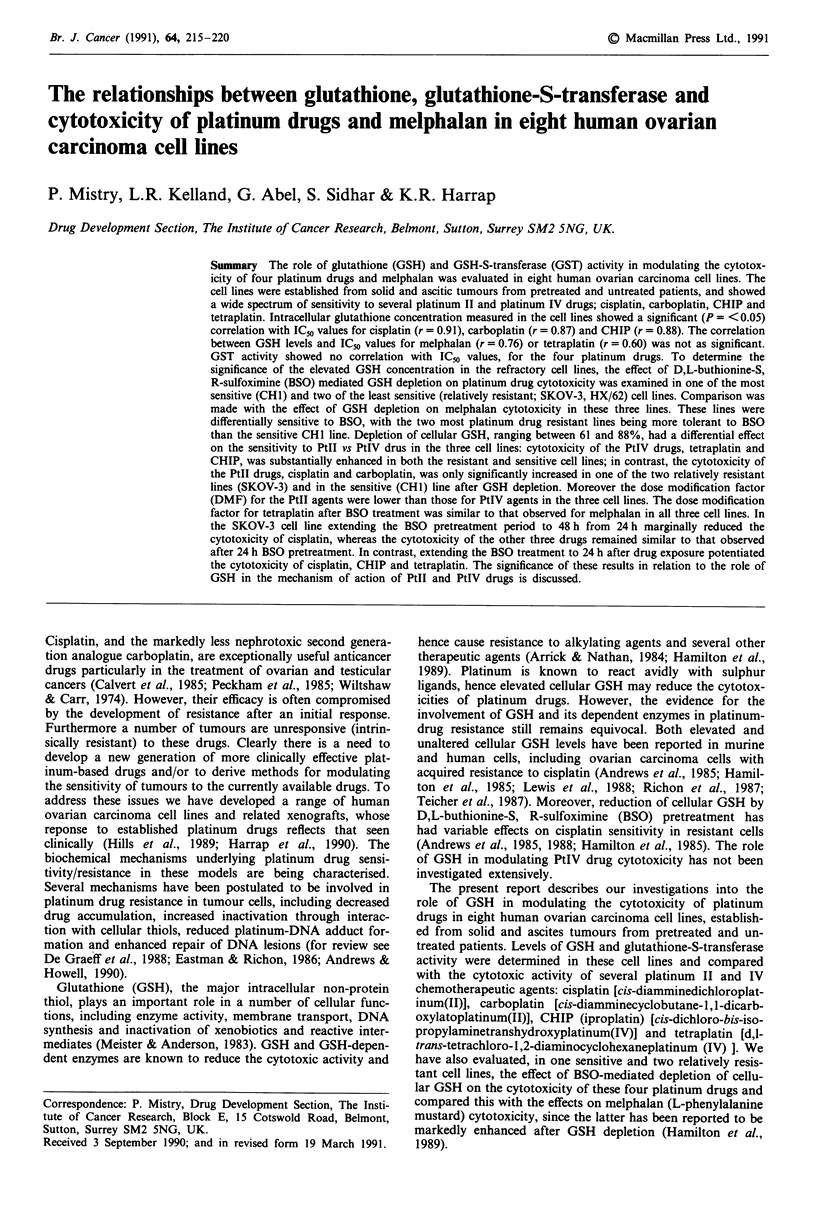

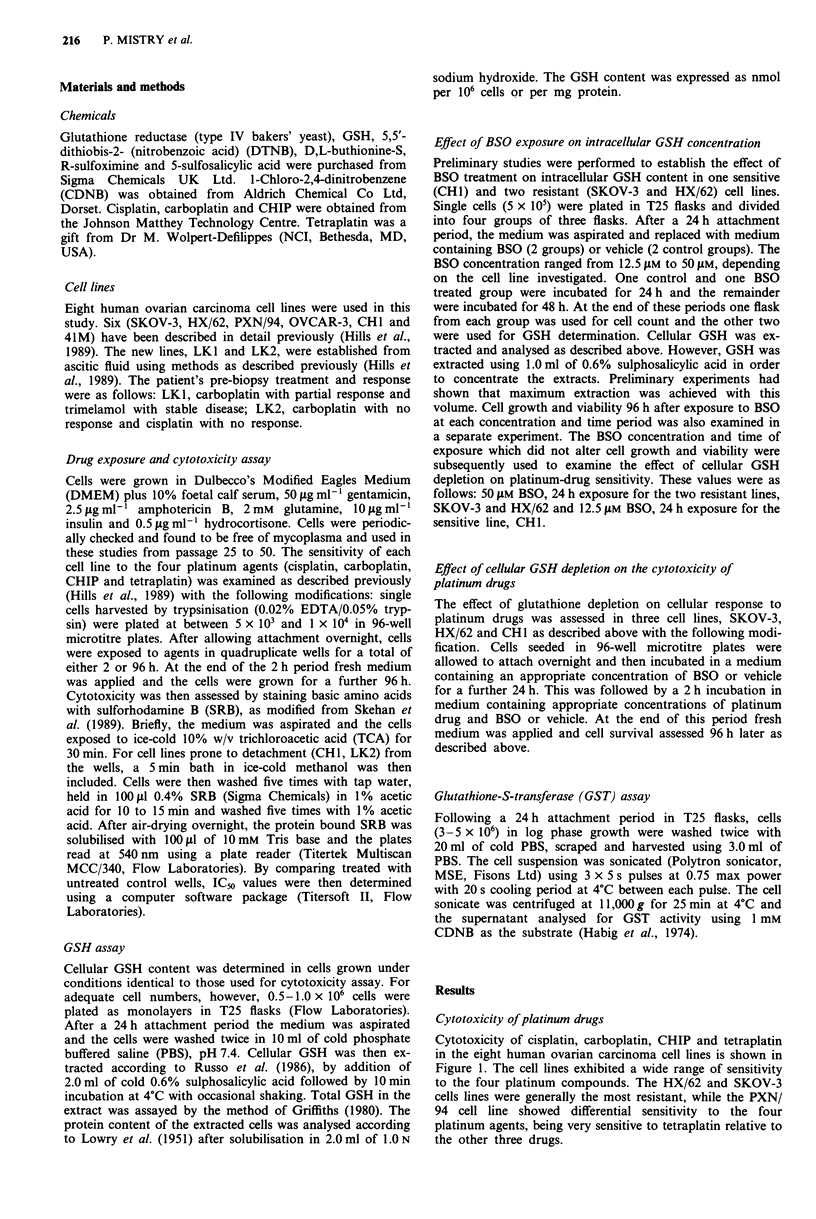

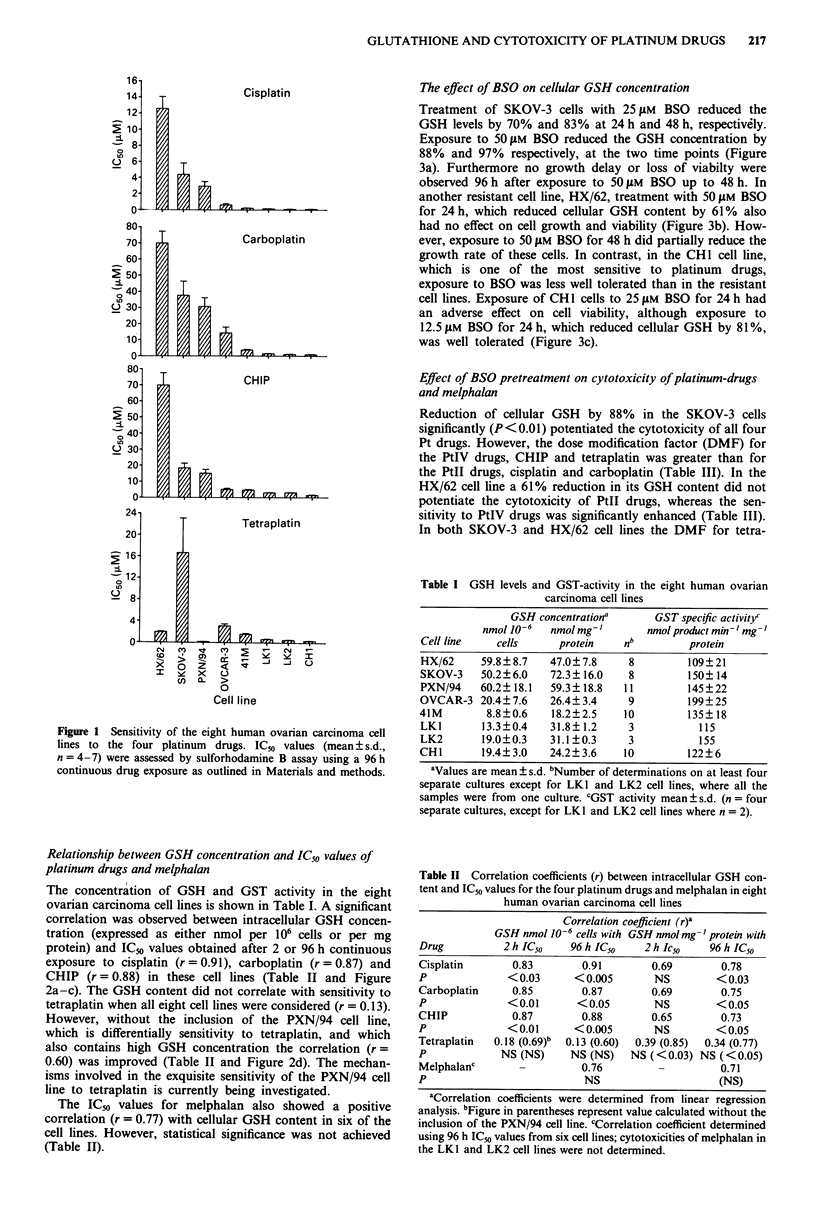

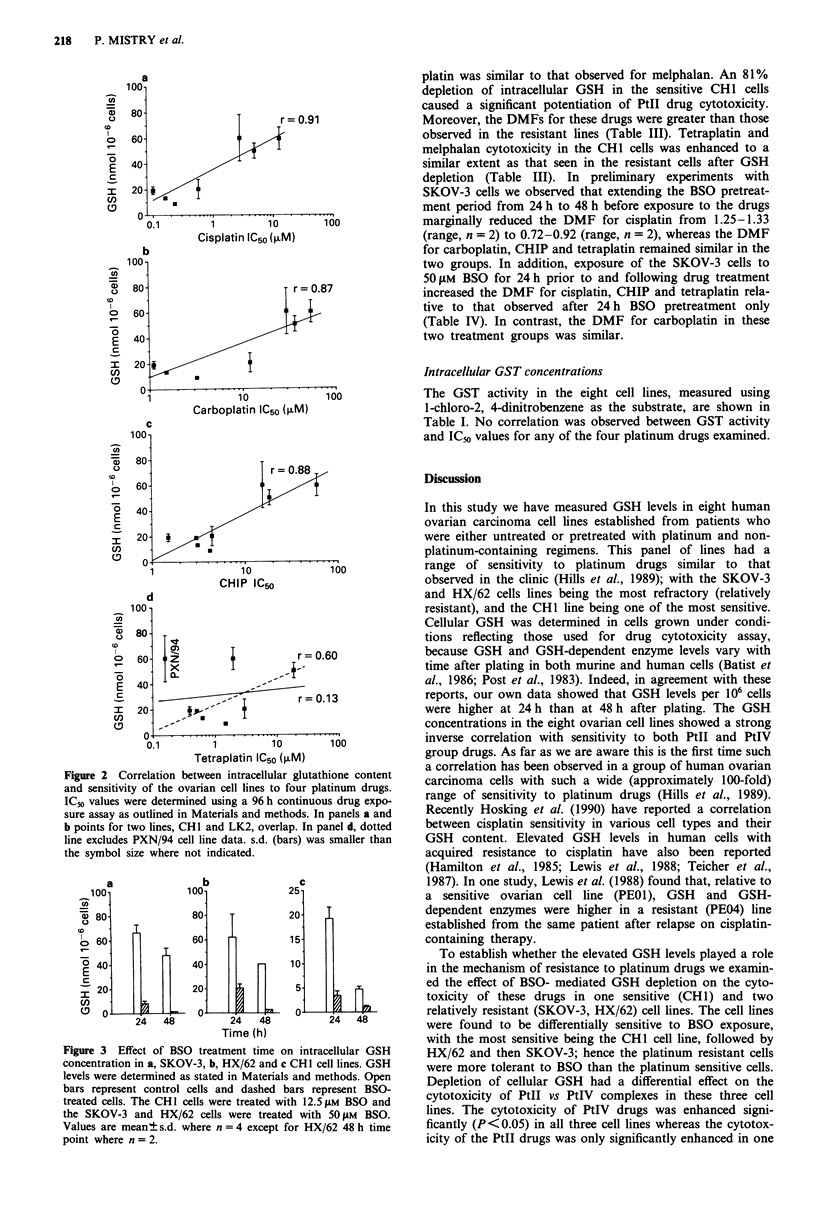

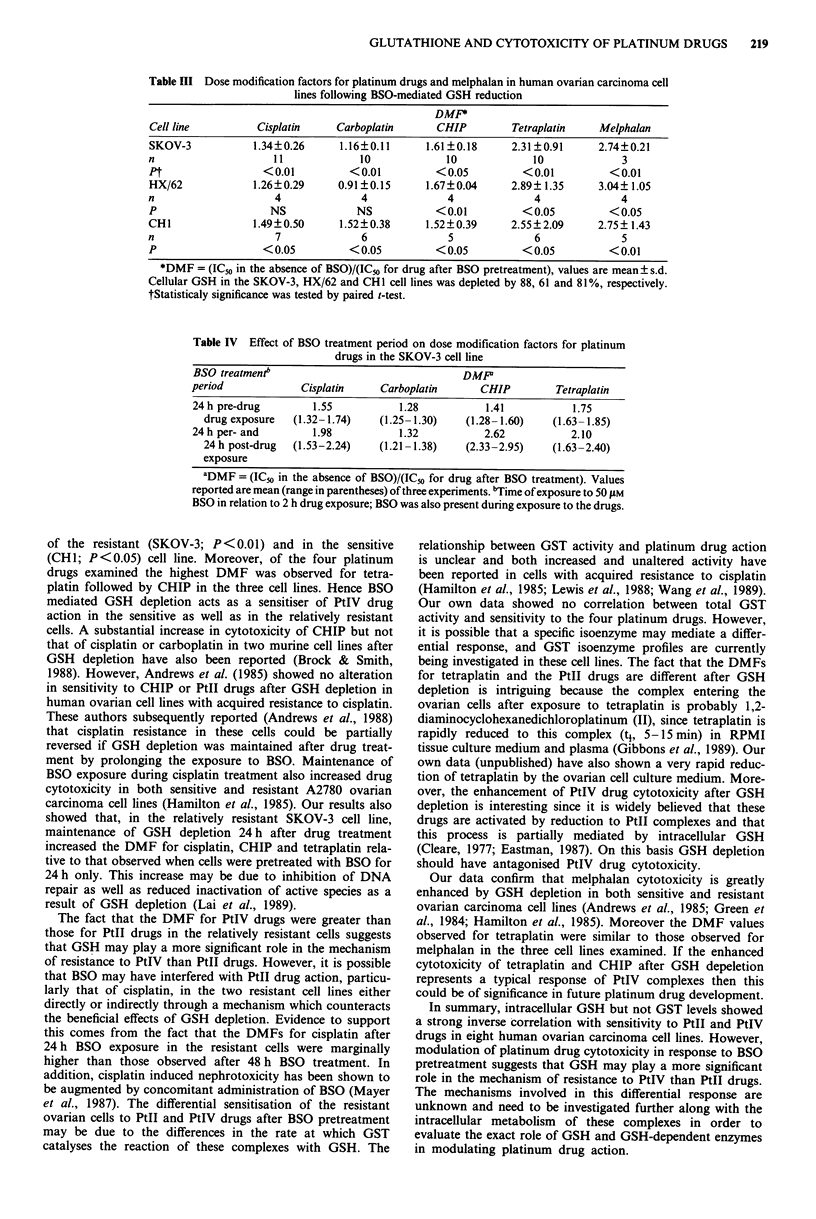

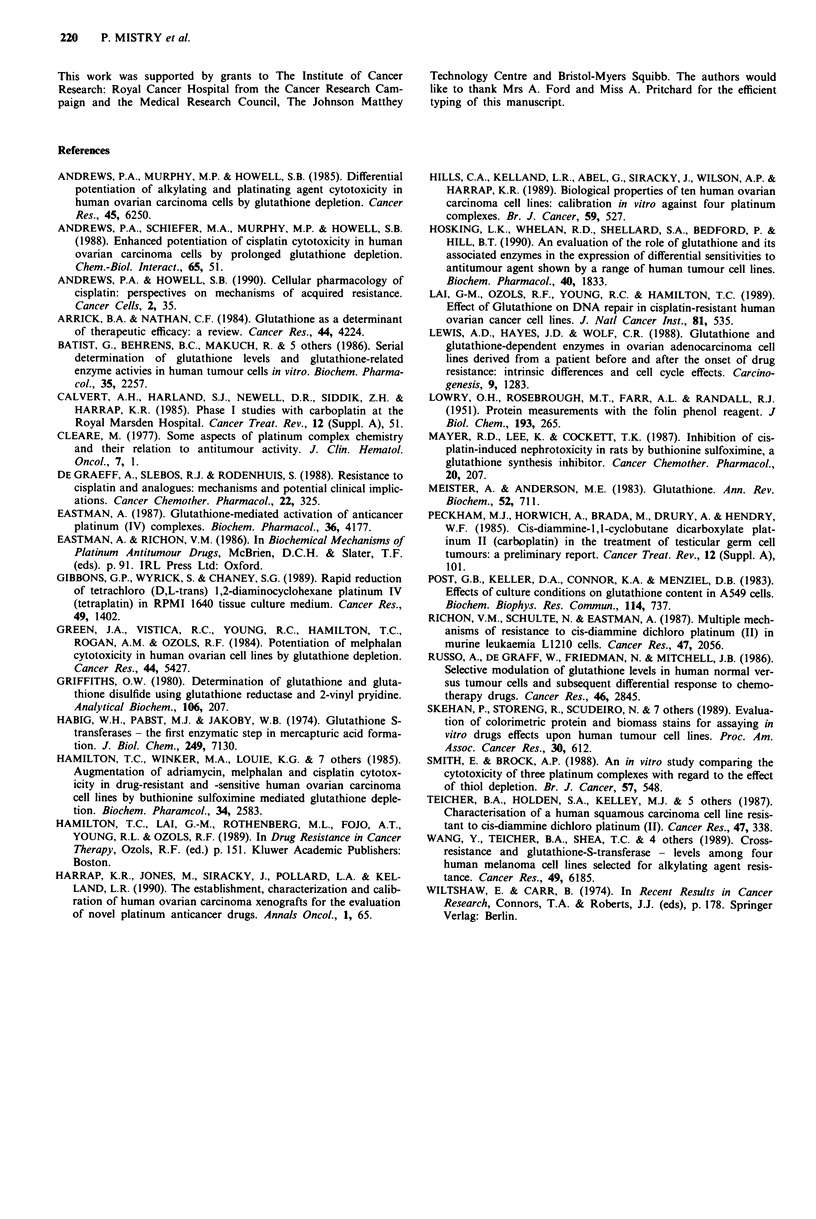


## References

[OCR_00885] Andrews P. A., Howell S. B. (1990). Cellular pharmacology of cisplatin: perspectives on mechanisms of acquired resistance.. Cancer Cells.

[OCR_00873] Andrews P. A., Murphy M. P., Howell S. B. (1985). Differential potentiation of alkylating and platinating agent cytotoxicity in human ovarian carcinoma cells by glutathione depletion.. Cancer Res.

[OCR_00879] Andrews P. A., Schiefer M. A., Murphy M. P., Howell S. B. (1988). Enhanced potentiation of cisplatin cytotoxicity in human ovarian carcinoma cells by prolonged glutathione depletion.. Chem Biol Interact.

[OCR_00890] Arrick B. A., Nathan C. F. (1984). Glutathione metabolism as a determinant of therapeutic efficacy: a review.. Cancer Res.

[OCR_00894] Batist G., Behrens B. C., Makuch R., Hamilton T. C., Katki A. G., Louie K. G., Myers C. E., Ozols R. F. (1986). Serial determinations of glutathione levels and glutathione-related enzyme activities in human tumor cells in vitro.. Biochem Pharmacol.

[OCR_00900] Calvert A. H., Harland S. J., Newell D. R., Siddik Z. H., Harrap K. R. (1985). Phase I studies with carboplatin at the Royal Marsden Hospital.. Cancer Treat Rev.

[OCR_00914] Eastman A. (1987). Glutathione-mediated activation of anticancer platinum(IV) complexes.. Biochem Pharmacol.

[OCR_00923] Gibbons G. R., Wyrick S., Chaney S. G. (1989). Rapid reduction of tetrachloro(D,L-trans)1,2-diaminocyclohexaneplatinum(IV) (tetraplatin) in RPMI 1640 tissue culture medium.. Cancer Res.

[OCR_00929] Green J. A., Vistica D. T., Young R. C., Hamilton T. C., Rogan A. M., Ozols R. F. (1984). Potentiation of melphalan cytotoxicity in human ovarian cancer cell lines by glutathione depletion.. Cancer Res.

[OCR_00940] Habig W. H., Pabst M. J., Jakoby W. B. (1974). Glutathione S-transferases. The first enzymatic step in mercapturic acid formation.. J Biol Chem.

[OCR_00952] Hamilton T. C., Lai G. M., Rothenberg M. L., Fojo A. T., Young R. C., Ozols R. F. (1989). Mechanisms of resistance to cisplatin and alkylating agents.. Cancer Treat Res.

[OCR_00945] Hamilton T. C., Winker M. A., Louie K. G., Batist G., Behrens B. C., Tsuruo T., Grotzinger K. R., McKoy W. M., Young R. C., Ozols R. F. (1985). Augmentation of adriamycin, melphalan, and cisplatin cytotoxicity in drug-resistant and -sensitive human ovarian carcinoma cell lines by buthionine sulfoximine mediated glutathione depletion.. Biochem Pharmacol.

[OCR_00964] Hills C. A., Kelland L. R., Abel G., Siracky J., Wilson A. P., Harrap K. R. (1989). Biological properties of ten human ovarian carcinoma cell lines: calibration in vitro against four platinum complexes.. Br J Cancer.

[OCR_00970] Hosking L. K., Whelan R. D., Shellard S. A., Bedford P., Hill B. T. (1990). An evaluation of the role of glutathione and its associated enzymes in the expression of differential sensitivities to antitumour agents shown by a range of human tumour cell lines.. Biochem Pharmacol.

[OCR_00989] LOWRY O. H., ROSEBROUGH N. J., FARR A. L., RANDALL R. J. (1951). Protein measurement with the Folin phenol reagent.. J Biol Chem.

[OCR_00977] Lai G. M., Ozols R. F., Young R. C., Hamilton T. C. (1989). Effect of glutathione on DNA repair in cisplatin-resistant human ovarian cancer cell lines.. J Natl Cancer Inst.

[OCR_00982] Lewis A. D., Hayes J. D., Wolf C. R. (1988). Glutathione and glutathione-dependent enzymes in ovarian adenocarcinoma cell lines derived from a patient before and after the onset of drug resistance: intrinsic differences and cell cycle effects.. Carcinogenesis.

[OCR_00994] Mayer R. D., Lee K. E., Cockett A. T. (1987). Inhibition of cisplatin-induced nephrotoxicity in rats by buthionine sulfoximine, a glutathione synthesis inhibitor.. Cancer Chemother Pharmacol.

[OCR_01000] Meister A., Anderson M. E. (1983). Glutathione.. Annu Rev Biochem.

[OCR_01004] Peckham M. J., Horwich A., Brada M., Drury A., Hendry W. F. (1985). cis-Diammine-1, 1-cyclobutane dicarboxylate platinum II (carboplatin) in the treatment of testicular germ-cell tumours: a preliminary report.. Cancer Treat Rev.

[OCR_01011] Post G. B., Keller D. A., Connor K. A., Menzel D. B. (1983). Effects of culture conditions on glutathione content in A549 cells.. Biochem Biophys Res Commun.

[OCR_01016] Richon V. M., Schulte N., Eastman A. (1987). Multiple mechanisms of resistance to cis-diamminedichloroplatinum(II) in murine leukemia L1210 cells.. Cancer Res.

[OCR_01021] Russo A., DeGraff W., Friedman N., Mitchell J. B. (1986). Selective modulation of glutathione levels in human normal versus tumor cells and subsequent differential response to chemotherapy drugs.. Cancer Res.

[OCR_01033] Smith E., Brock A. P. (1988). An in vitro study comparing the cytotoxicity of three platinum complexes with regard to the effect of thiol depletion.. Br J Cancer.

[OCR_01038] Wang Y. Y., Teicher B. A., Shea T. C., Holden S. A., Rosbe K. W., al-Achi A., Henner W. D. (1989). Cross-resistance and glutathione-S-transferase-pi levels among four human melanoma cell lines selected for alkylating agent resistance.. Cancer Res.

[OCR_00909] de Graeff A., Slebos R. J., Rodenhuis S. (1988). Resistance to cisplatin and analogues: mechanisms and potential clinical implications.. Cancer Chemother Pharmacol.

